# Supplementary material for: Physical activity status among adolescents in Southern Ethiopia: A mixed methods study
Source: PLoS One. 2023 Nov 9;18(11):e0293757. doi: 10.1371/journal.pone.0293757 (PMC10635445; doi:10.1371/journal.pone.0293757)
Supplement: Attachment — Submitted filename: Response to Reviewers.docx [file pone.0293757.s002.docx]

| **Responses to editor’s comment**  **Editor’s comments, suggestions and questions are considered and carefully revised the manuscript as per the suggestions and comments. We are very thankful to the potential editor for suggestions and comments, which substantially improved the manuscript** | |
| --- | --- |
| **Editors comments** | **Responses** |
| 1. Please ensure that your manuscript meets PLOS ONE's style requirements, including those for file naming. The PLOS ONE style templates can be found at   https://journals.plos.org/plosone/s/file?id=wjVg/PLOSOne_formatting_sample_main_body.pdf and  https://journals.plos.org/plosone/s/file?id=ba62/PLOSOne_formatting_sample_title_authors_affiliations.pdf | Thank you, dear editor, we have re-written the manuscript based on PLOS ONE's style templates as per suggestion you have given **(Line ……)** |
| 2. PLOS requires an ORCID iD for the corresponding author in Editorial Manager on papers submitted after December 6th, 2016. Please ensure that you have an ORCID iD and that it is validated in Editorial Manager. To do this, go to ‘Update my Information’ (in the upper left-hand corner of the main menu), and click on the Fetch/Validate link next to the ORCID field. This will take you to the ORCID site and allow you to create a new iD or authenticate a pre-existing iD in Editorial Manager. Please see the following video for instructions on linking an ORCID iD to your Editorial Manager account: https://www.youtube.com/watch? | Thank you dear editor. The corresponding author have included ORCID iD.  The ORCID iD is  https://orcid.org/0000-0002-1433-1332 |
| 3. Please review your reference list to ensure that it is complete and correct. If you have cited papers that have been retracted, please include the rationale for doing so in the manuscript text, or remove these references and replace them with relevant current references. Any changes to the reference list should be mentioned in the rebuttal letter that accompanies your revised manuscript. If you need to cite a retracted article, indicate the article’s retracted status in the References list and also include a citation and full reference for the retraction notice. | Thanks dear editor we have revised our manuscript as per your comment and suggestion. |
| **1^st^ reviewer comments** | **Responses** |
| 1. In the introduction section, the authors provided similar information repeatedly; please provide each concept in concise AND ONCE. | Thank you dear reviewer for your comments. We have revised the manuscript as per your comments. We have made revisions in the introduction part of the manuscript. Similar sentences were come together in order to have consistence meaning. **Line 46-48, 56-58** |
| 2. Similarly, the authors didn't mention the factors and barriers associated with PA please provide these factors in the introduction section. | Thank you very much dear reviewer for your comments and suggestions. In the article we have included some factors associated with physical activity level like age, sex, depression and others. **Line 64-66, 72-74** |
| 3. Regarding the methods, based on the information given in the background section, the authors emphasized the importance of physical environment for PA, as a result, its assumed that the authors would have stratified the school children based on the school area (towns), while they simply focused on gender as a source of variation. Any clarification? | Thanks dear reviewer for your comments. As we have stated in the study setting section of the method **(line 143-145)**, we have selected urban then rural because of the risk, we have included this as “urban dwellers are exposed more to sedentary behaviours as a consequence of the use of new technologies in Africa (TV, Movies, Smart phones and Social Medias,etc)”. **Line 144-146.** |
| 4. The exclusion criteria are not clearly stated. Moreover, what's the impact of individuals who do not provide consent on the recruitment process and how they will be handled? | Thanks dear reviewer for your comments. As we have mentioned in the ethical consideration sub-section of the method. After explaining the aim of the study, and their full voluntariness to participate in the study, written informed consent from the participants, school administrators and parents were taken. If they were not willing to participate in the study and not gave consent, they will be excluded from the study (**Line 279-285)**. We have stated in the result section, there are about 24 participants that were excluded from the study because of their refusal (**line 378-380**). As per your comment we have included exclusion criteria in the revised version of the manuscript. **Line 157-158** |
| 5. Regarding the sampling procedure, the author’s selected three out of FIVE government high schools RATHER than two OR four might be, justify it? | Thank you dear reviewer for your comments. In order to have adequate and representative sample it was advisable to take more diverse schools in the study. As it was mentioned in the sampling procedure sub-section of the method section (**line 171-178**) we have included at least half of the schools from both government and private schools, which intern improves the representativeness of the study. |
| 6. Overall, the manuscript will benefit from proper proofreading and editing because I cannot list all the language and punctuation mistakes in the text | Thank you dear reviewer for your comments. We have revised the manuscript as per your comments starting from background up to conclusion. **Line 46-51, 54-59, 67-74, 100-117, 125-176, 212, 215-216, 220, 230-260, 332-356, 378-413, 460-480, 523-542, 594-613,671-688.** |
| **2^nd^ reviewer comments** | **Response** |
| 1. Dependent variable measurement and quantitative data collection needs further explanation | Thank you for the valuable suggestion. We have elaborated more details about outcome variable measurement and how the quantitative data collection were done. In the Method section under the three sub sections ‘data collection tool and procedure, variable of the study and measurements’ **(lines 203-206, 222-223, 227-242).** |
| 2. The use of space between the end of the sentence and a reference must be carefully corrected throughout the manuscript. | Thank you very much dear reviewer for your comments. We have considered your comments and corrected the use of punctuations in the revised version of the manuscript. **Line 46, 49, 51, 56, 66, 68, 76, 82,** |
| 3. The last three lines on the sample size determination needs correction. | Thanks dear reviewer for your constructive comments. We have revised the last three lines of the sample size calculation as per your comments as “For the qualitative data, six focus group discussions (FGDs) (the number of FGDs was determined based on idea saturation) were formed in groups of male and female students. Each of the groups consisted of 8 to 12 members.” **Line 168-172** |
| 4. The use of multi-centered is not appropriate as long as the data from the two cities and high schools is not treated in the analysis. I.e., the effect of being an adolescent from high schools in the two cities is not included the multiple regression analyses. | Thanks dear reviewer for your comments. We have removed the phrase “multi-centre “from the title of the study. Moreover, we did not adjust the town variable in the regression analysis for we assumed that the two towns may not have a difference in PA activity culture as they are both zonal towns with similar or comparable infrastructure for sport and other activities. Previous literatures have also indicated the factors as emanating from individual circumstances and behaviours and playing at the level of adolescents and their school environment. |
| 5. How is the actual data collection conducted? If it was a self-administered, why is the facilitators read each question being at the stage of the classroom? | Thank you for your insightful comment. Indeed, the way the data has been obtained is stated as self-administered in the sense that students filled their response by their own. Facilitators were there just to ensure the quality of data since some of the students were young and minor who may not give weight to the purpose of the study. Thus, to make everyone get the same information and understanding, the facilitators read each question to the class so that everyone can fill his or her answer after listening to the questions. Therefore, we can not strictly say that this is interviewer administered. We believe that this is ethical as the facilitators were away on the stage and asking the students only to hear to them. |
| 6. Physical education is part of their school curriculum which involves different level of physical activity on weekly basis. Is the PA activity that adolescents perform while at school considered in the PA measurement? Depending on the number of Physical education session, students may involve in 50 to 100 minutes. | Thank you for raising this pertinent question. In the context of this study, students had no physical activity session as the data was collected at a time when schools opened after the COVID-19 lock-down when strict physical distancing was in place. They were only given a theoretical class on few critically important subjects in a sequential manner. Had the situation was not that too, the study does not preclude PA done in school environments as the intent is as to how much students were active wherever they are which we have tried to incorporate on item 8 of our modified version tool. However, the very nature of the PAQ-A does not ask this information which can be considered as a limitation of the tool as questions are more of focusing to obtain information on mobility or activity distributed over the day rather than cumulative daily hours of PA. |
| 7. Instead of removing the three variables from regression because of non-linearity, why not you transformed PA. | Thank you for your insightful query. Since PA was measured as a composite variable, we were concerned with its transformation and the subsequent nuisance in interpretation of the findings. |
| **Additional corrections made by the authors** | |
| 1. We didn’t include the title recommendation in the old manuscript but in the revised manuscript, we included the title ‘Recommendation.’ **Line 676** | |
